# Genetic profile of non‐small cell lung cancer (NSCLC): A hospital‐based survey in Jinhua

**DOI:** 10.1002/mgg3.1398

**Published:** 2020-07-12

**Authors:** Xianguo Chen, Bo Xu, Qiang Li, Xiaoyi Xu, Xianshuai Li, Xia You, Zhaonan Yu

**Affiliations:** ^1^ Department of Thoracic Surgery Jinhua Municipal Central Hospital Jinhua Hospital of Zhejiang University Jinhua China; ^2^ Hangzhou D.A. Medical Laboratory Hangzhou China

**Keywords:** clinical characteristics, genetic profile, nonsmall cell lung cancer, targeted sequencing

## Abstract

**Background:**

We describe the clinical features, genetic profile, and their correlation in NSCLC patients.

**Methods:**

A total of 256 Chinese patients with NSCLC were enrolled in this study. NGS‐based genomic profiling of major lung cancer‐related genes was performed on formalin‐fixed paraffin‐embedded tumor samples.

**Results:**

Of 256 patients with NSCLC, 219 were adenocarcinoma and most of them were in the early stage. Among patients, 63.3% patients have more than two gene mutations. By analyzing variant allele frequency (VAF), we found that the median VAF has significant differences between squamous cell carcinoma and adenocarcinoma, as well as early stage and advanced stage. The frequency of mutations in *EGFR*, *MET*, and *RET* were significantly higher in nonsmokers than in smokers. Besides, Pearson correlation analysis found that *ALK*, *BRAF,* and *MET* mutations had a strong correlation with age. Notably, higher frequencies of *ALK* and *BRAF* alterations were associated with younger age, while more frequent *MET* mutations appear in the patients at age 55 or older.

**Conclusion:**

More unique features of cancer driver genes in Chinese NSCLC were identified by next‐generation sequencing. These findings highlighted that it is necessary to carry out targeted detection according to different clinical features for NSCLC.

## Graphical abstract

We described clinical characteristics and genetic profiles of 256 patients with non‐small cell lung cancer. We found that the median variant allele frequency (VAF) in adenocarcinoma was significantly lower than those in squamous cell carcinoma and the median VAF was significantly higher in stage II‐IV than in stage I. Besides, we found that ALK and BRAF mutations had a strong correlation with younger age.

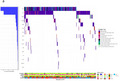

## 1 INTRODUCTION

Lung cancer remains the most common cancer. It is reported that there were 2.1 million new lung cancer cases (11.6% of the total), and 1.77 million cancer death (18.4% of the total) in 2018 (Bray et al., 2018). According to China Center for Disease Control and Prevention in 2019, there were 787,000 new lung cancer cases and 631,000 cancer related death in China (Zheng et al., 2019). Nonsmall cell lung cancer (NSCLC) is the most common cause of cancer‐related mortality worldwide, approximately 85% of patients being NSCLC (Reade & Ganti, 2009). Lung adenocarcinoma (LUAD) is the most common histologic type of NSCLC, which cigarette smoking as the major cause (Behera et al., 2016; Kudo et al., 2015; Travis et al., 2011).

There are high rates of somatic mutation and genomic rearrangement in NSCLC. Owing to studies of somatic mutation activation in NSCLC patients, we already know that a series of driver genes, including *EGFR*, *BRAF*, *KRAS*, *MET*, *ALK*, and *ROS1* were the most frequent mutation genes in LUAD (Hirsch et al., 2017; Shi et al., 2016; Cancer Genome Atlas Research Network 2014). The latest version of the National Comprehensive Cancer Network (NCCN) guidelines for NSCLC recommends that, in addition to detecting *EGFR*, *KRAS*, *BRAF*, *HER2* mutations, *MET* amplification/exon 14 skipping mutations and gene rearrangements involving *ROS1*, *RET*, *ALK*, etc., *NTRK* gene fusion should also be genetically tested. Due to the discovery of driver mutations, many patients could become eligible for targeted therapy. The gene mutations in lung cancer have different results among different countries, for example, the frequency of *EGFR* mutation of Asian female never‐smokers higher than female never‐smokers of the west countries. So, it is necessary to understand comprehensive profiling of genetic mutations in the Asian population to guide diagnosis and therapies for NSCLC.

In this study, we used a well‐validated assay to perform 18 gene mutations profiling on tumor specimens from a community hospital‐based NSCLC patients in Jinhua city. We also describe the correlation of age, gender, smoking, tumor stage, histological type with genetic mutations.

## 2 MATERIALS AND METHODS

### 2.1 Patients

This study enrolled 256 patients with NSCLC who underwent surgery at Jinhua Municipal Central Hospital from July 2018 to May 2019. All patients medical record was retrospectively collected (Table 1). Pathological slides were from tissue specimens of surgical resection. The histological diagnosis of all sections was evaluated by experienced pathologists to ensure that the tumor cell ratio was greater than 20%. The ethical review was approved by the Ethics Committee of JinHua Municipal Central Hospital. Informed consent was signed by each patient participating in the study prior to the trial.

**Table 1:** Clinical characteristics of 256 patients with NSCLC with next‐generation sequencing assay

Characteristics	No. (%)	Adenocarcinoma (%)	Squamous cell carcinoma (%)
Gender			
Male	106 (41.4%)	78 (30.5%)	13 (5.1%)
Female	150 (58.6%)	141 (55.1%)	1 (0.4%)
Age			
≤55 years	74 (28.9%)	68 (26.6%)	0
55–70 years	132 (51.6%)	117 (45.7%)	10 (3.9%)
>70 years	40 (15.6%)	33 (12.9)	2 (0.8%)
Unknown	10 (3.9%)	/	/
Smoking history			
Yes	51 (19.9%)	36 (14.1%)	8 (3.1%)
No	205 (80.1%)	183 (71.5%)	6 (2.3%)
Histological type			
Adenocarcinoma	219 (85.5%)	/	/
Squamous cell carcinoma	16 (6.3%)	/	/
Adenosquamous carcinoma	1 (0.4%)	/	/
Unknown	20 (7.8%)	/	/
Tumor stage			
I	175 (68.4%)	169 (66.0%)	5 (2.0%)
II‐IV	43 (16.8%)	6 (10.9%)	8 (3.1%)
Unknown	38 (14.8%)		

### 2.2 DNA extraction

According to the manufacturer's recommendations, the formalin fixed paraffin‐embedded (FFPE) sample genomic DNA were extracted using QIAamp^®^ DNA FFPE Tissue Kit (product number: 56404). The purity of the extracted product was detected by Nanodrop2000, and the concentration was determined using Nanodrop2000 (Thermo) and Qubit 3.0 (Invitrogen). Then, all qualified samples were used for subsequent experiments.

### 2.3 Library preparation and next generation sequencing

Genomic DNA was randomly sheared into fragments of 150–200 bp in length by Covaris. The library of qualified genomic DNA was subjected to construct, and the sequencing libraries were generated using SureSelect^XT HS^ Target Enrichment System. The library quality (concentration and insert size) was assessed on the Qubit 3.0 Fluorometer （Invitrogen） and Agilent Bioanalyzer 4200 system. Then, the library was diluted to 1.4 pM. Finally, targeted sequencing was carried out using the Illumina Nextseq500 platform (Illumina) and 150 bp paired‐end reads were generated. This targeting panel contains 18 genes with single nucleotide variate (SNV), insertions/deletions (InDel), copy number variation (CNV), and gene fusions. The average sequencing depth was >1000X. The preparation of libraries and next generation sequencing were performed by Dian Diagnostics, Hangzhou.

### 2.4 Sequence alignment and variant calling

Use the fastp software to perform the reads filtering on the raw data of the FastQ format generated by the Illumina platform and perform comprehensive quality control by fastQC (http://www.bioinformatics.babraham.ac.uk/projects/fastqc/) on the data to obtain clean data (Chen, Zhou, Chen, & Gu, 2018). Then, clean data were aligned to reference human genome (UCSC hg19) by BWA software to capture the aligned bam files (Li & Durbin, 2009). The SAM tools and Picard (http://broadinstitute.github.io/picard/) software were implemented to rearrange and correct the bam files to obtain the final bam file (Li et al., 2009). The somatic variations were detected using Mutect software (Cibulskis et al., 2013). CNV kit was applied to detect somatic CNVs (Talevich et al., 2016). Fusion gene detection was performed using Illumina's manta tool (Chen and Schulz‐Trieglaff et al., 2016). Finally, we use the ANNOVAR and snpEff software to annotate vcf files with databases such as ensemble, RefSeq and 1000G (Cingolani et al., 2012; Wang et al., 2010). Here, it should be specially pointed out that variant allele frequency（VAF）was corrected by the proportion of tumor cells in this study, which further ensured the correctness of the mutation frequency to some extent.

### 2.5 Statistical analysis

Statistical analysis was carried out with R. Fisher's exact test and chi‐square test were used to correlate gene mutations with clinical features (age, gender, smoking, Histological type). Statistical significance was *p* < .05.

## 3 RESULTS

### 3.1 Clinical characteristics of nonsmall‐cell lung cancer patients

From July 2018 to May 2019, we consecutively collected 256 patients with NSCLC who underwent surgery at Jinhua Municipal Central Hospital. Of the 256 patients, 106 were male (41.4%), 150 were female (58.6%). 51 were smokers, 205 were non‐smokers. Among these patients, 74 were aged less than 55 years, accounted for 28.9% of the total cases; 172 were aged more than 55 years, accounted for 67.2% of the total cases. Patients in different ages and sex had similar histological classification (adenocarcinoma was the most prevalent). Regarding histological subtypes, adenocarcinoma was the most common subtype (85.5%, 219/256) and adenosquamous carcinoma was the least common subtype (0.4%, 1/256), while squamous cell carcinoma was 6.3% (16/256). In addition, 7.8% of the cases were of uncertain subtypes. There were 68.4% (175/256) patients with stage I disease, 16.8% (43/256) with stage II‐IV disease, and 14.8% (38/256) with unknown stage disease. The demographics and clinical characteristics of all 256 patients were summarized in Table 1.

### 3.2 Somatic DNA alterations

**Figure 1:**
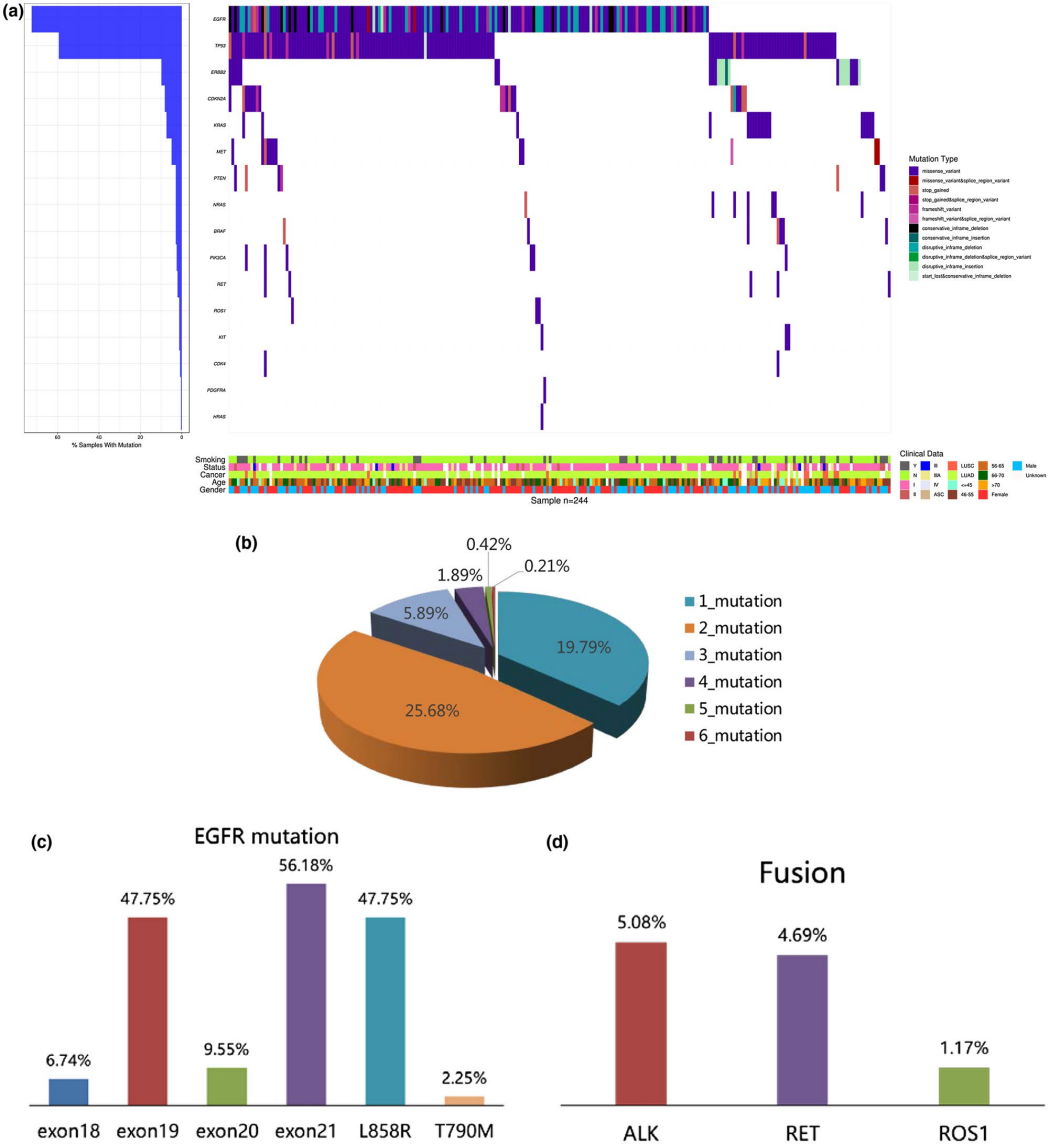
The genetic profile of nonsmall cell lung cancer (a) For 244 patients (each column), altered genes (rows) with mutations are shown. The percentage of samples with a mutation is noted at the left. The type of genetic mutation is presented in the middle. Clinical information is presented at the bottom. (b) Distribution of altered gene numbers in 256 patients with nonsmall cell lung cancer. (c) Bar chart showing the frequency of *EGFR* mutation in different subtypes of all NSCLC

A total of 256 patients with NSCLC were included in the study for genetic testing. Among all mutation types, the most common type of mutation was SNV (78.5%), followed by INDEL (12.5%), fusion gene (7%), and CNV (2%). In the 256 samples tested, one or more genomic alterations were identified in all the patients (Figure 1b), 94 (36.7%) patients having one mutation, 122 (47.7%) two mutations, and 40 (15.6%) more than two mutations. The landscape of driver mutations had been shown in Figure 1a. The most frequently mutated genes were *EGFR* (178 of 256, 69.5%), *TP53* (145 of 256, 56.6%), *ERBB2* (25 of 256, 9.8%), *CDKN2A* (20 of 256, 7.8%) and *KRAS* (18 of 256, 7.0%), which had all been reported as well‐known driver genes of NSCLC. The next frequently mutated genes included *MET* (4.7%), *PTEN* (2.7%) and *ROS1* (2.7%), *BRAF* (2.7%), *NARS* (2.7%), and *CDK4* (2.0%). The majority of mutations appeared in *EGFR*, *ERBB2* and *TP53*. The most common gene rearrangements were *ALK* (5.1%), *RET* (4.7%), and *ROS1* (1.6%). In total, 69.5% of the 256 patients were identified to have *EGFR* mutations, which excluded 1.9% amplification of *EGFR*. For *EGFR* mutations, we found that its mutations mainly occurred in exon19 and exon21 (Figure 1c), which was consistent with the results of many other studies (Gou & Wu, 2014). In addition, we had found several amplifications of *EGFR*, *CDK4*, *CDK6*, *MET* and *ERBB2*. Moreover, we found that three fusion genes, *ALK* (5.08%), *RET* (4.69%), and *ROS1* (1.17%) (Figure 1d), and these fusion genes were more likely to occur in early stage patients.

### 3.3 Mutation distribution of all patients

**Figure 2:**
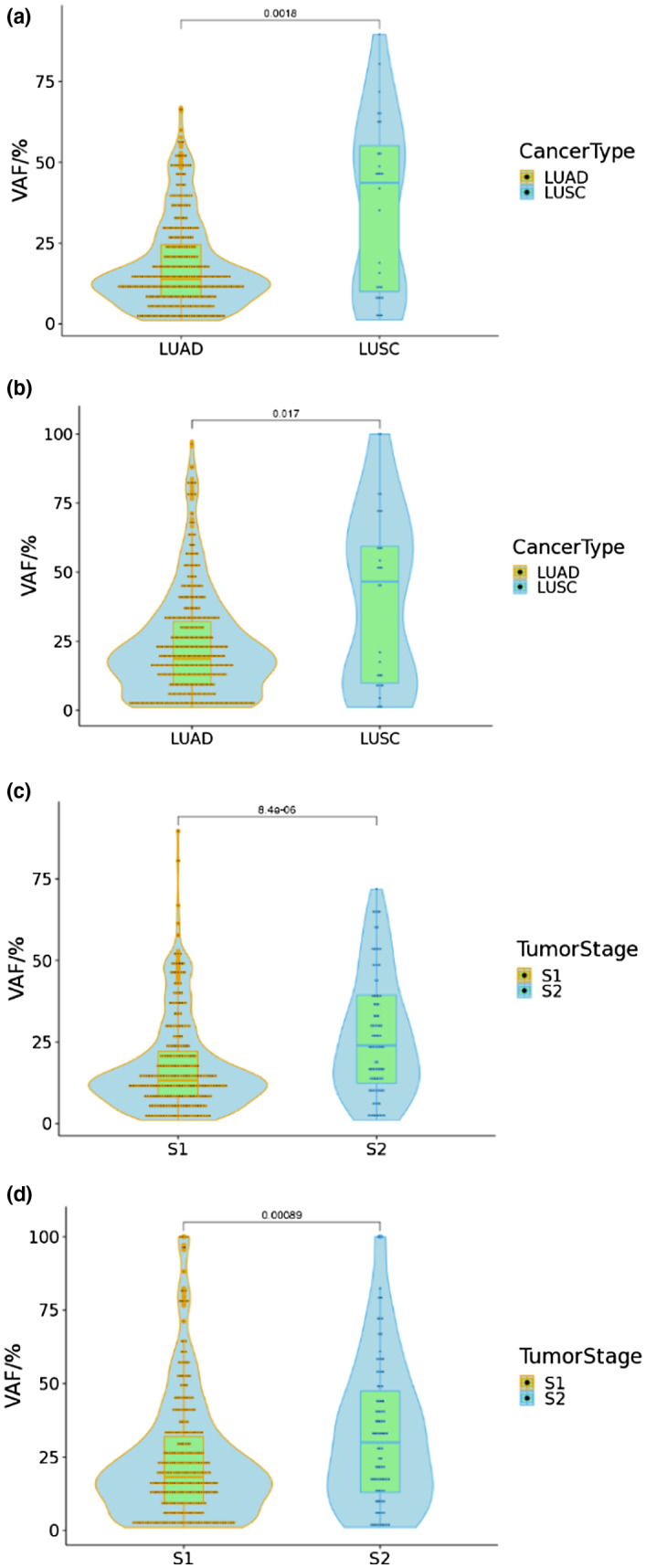
Comparison of variant allele frequencies in tumor type and tumor stage (a) Comparison of variant allele frequencies of LUAD and LUSC with uncorrected data. (b) Comparison of variant allele frequencies of patients with stage I and stage II‐IV with uncorrected data. (c) Comparison of variant allele frequencies of LUAD and LUSC with corrected data. (d) Comparison of variant allele frequencies of patients with stage I and stage II‐IV with corrected data. LUAD: Lung adenocarcinoma. LUSC: Lung squamous cell carcinoma. S1: patients with stage I. S2: patients with stage II‐IV. Statistical significance was defined as p＜0.05

We collected clinical information (including gender, histological type, smoking history, age, and tumor stage) of these patients, then we determined the relationship between clinical features and genetic mutations. We compared the frequency of mutations between lung adenocarcinoma and lung squamous cell carcinoma, in addition to this, compared between stage I and stage II or higher. These data were corrected and uncorrected. Additionally, in the absence of uncorrected data we found that the median VAF was significantly lower in adenocarcinoma than in squamous carcinoma (Figure 2a, *p* = .0018). The median VAF of adenocarcinoma was 13.8% and squamous carcinoma 43.6%. Similarly, we compared the VAF of patients with stage I and stage II or higher and found that patients with stage II‐IV had significantly higher VAF than patients with stage I (Figure 2c, *p* = 8.4e‐06). The median VAF of patients with stage I was 18.1%, and the patients with stage II‐IV was 29.9%. We conducted a statistical analysis with and without corrected data and found a similar trend, either between the lung squamous carcinoma and lung adenocarcinoma (Figure 2b, *p* = .017) or between stage I patients and stage II‐IV patients (Figure 2d, *p* = .00089). This suggested that there is indeed a significant difference in the VAF between lung squamous cell carcinoma and lung adenocarcinoma, as well as early and advanced patients.

**Figure 3:**
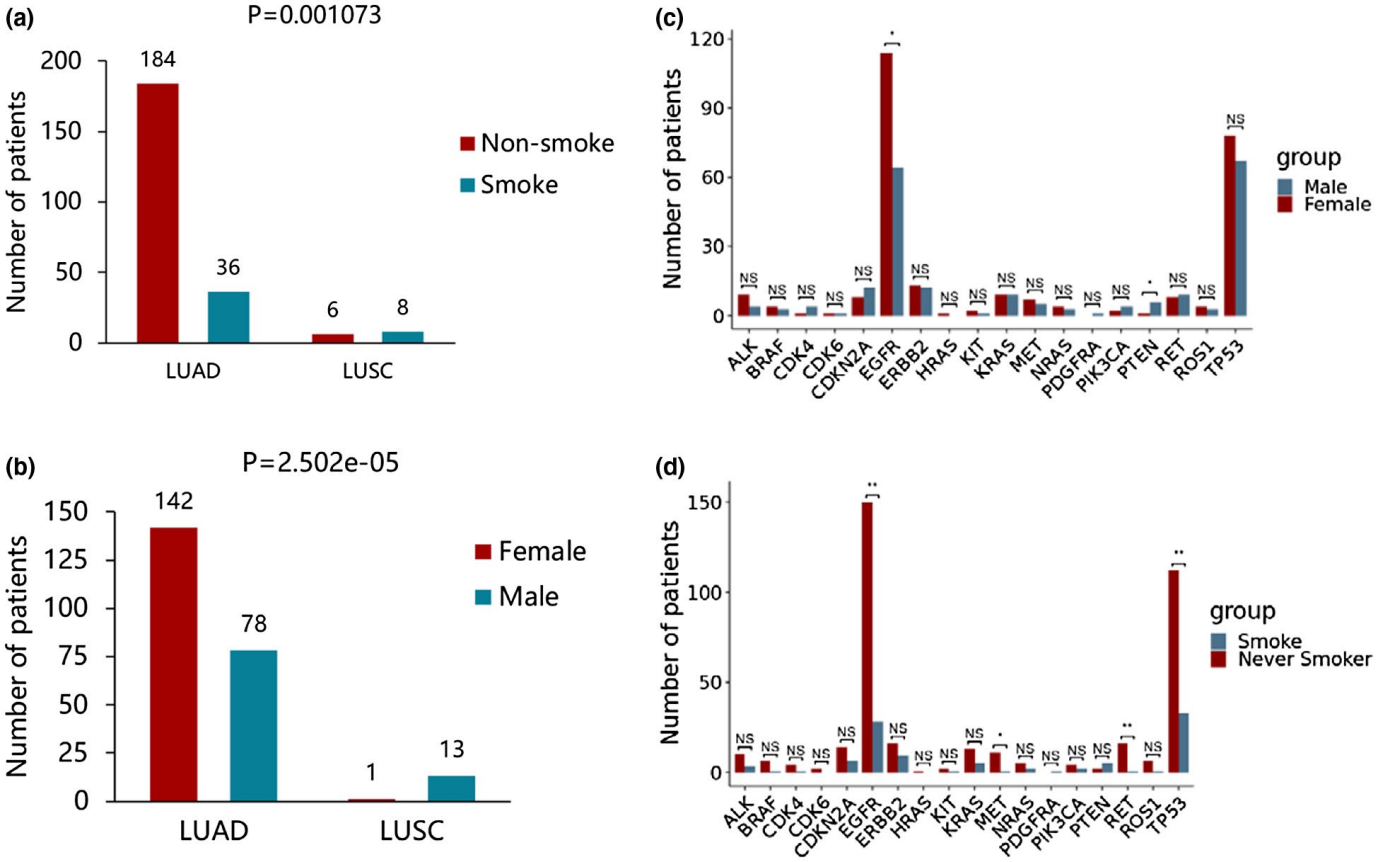
Comparison of variant allele frequencies in tumor type and tumor stage (a) Comparison of variant allele frequencies of LUAD and LUSC with uncorrected data. (b) Comparison of variant allele frequencies of patients with stage I and stage II‐IV with uncorrected data. (c) Comparison of variant allele frequencies of LUAD and LUSC with corrected data. (d) Comparison of variant allele frequencies of patients with stage I and stage II‐IV with corrected data. LUAD: Lung adenocarcinoma. LUSC: Lung squamous cell carcinoma. S1: patients with stage I. S2: patients with stage II‐IV. Statistical significance was defined as p＜0.05

We also compared the effects of gender and smoking history on the distribution of genetic mutations in patients under different histological types. The results were presented in Figure 3, there was a significant difference in the proportion of lung squamous cell carcinoma and lung adenocarcinoma between smokers and nonsmokers (Figure 3a, *p* = .001073). Similarly, a significant difference in the proportion of lung squamous cell carcinoma and lung adenocarcinoma between male and female was observed (Figure 3b, *p* = 2.502e‐05). Among patients with lung adenocarcinoma, nonsmokers were higher than smokers, females were more than males. However, in lung squamous cell carcinoma, the results were reversed. Although the number of patients was smaller, the results of this study were consistent with previous studies (Sun et al., 2007).

**Figure 4:**
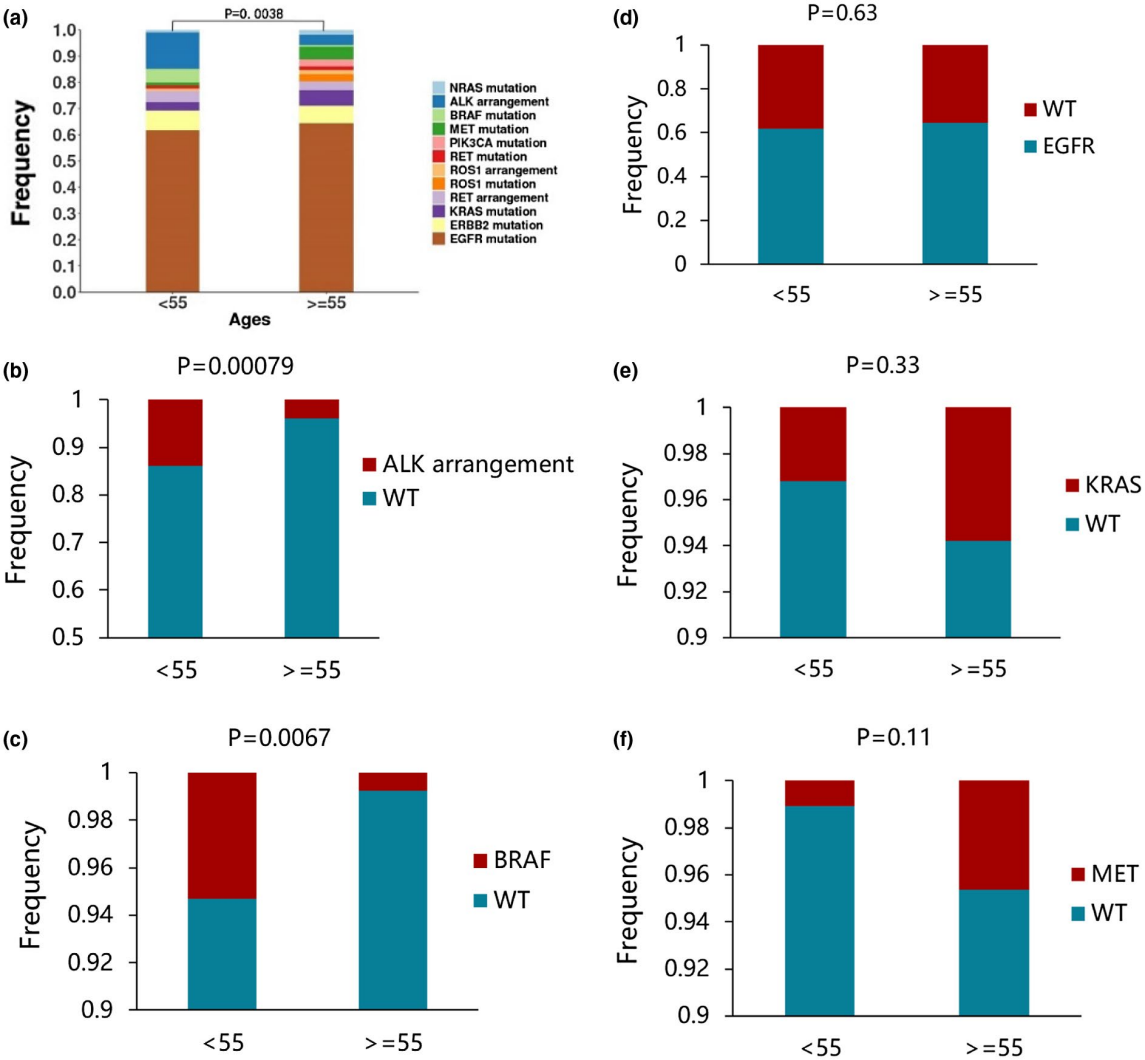
The distribution of representative targeted genetic alterations between the younger and older patients with lung adenocarcinoma (a) The genetic profiles in different age groups of patients with nonsmall‐cell lung cancer. 246 patients with NSCLC patients were enrolled, and tissue sample were analyzed by NGS assays. (b‐f) The distribution of representative targeted genetic alterations between the younger and older patients. (b) *ALK* arrangements, (c) *BRAF* mutations, (d)*EGFR* mutations, (e) *KRAS* mutations, (f) *MET* mutations. Statistical significance was defined as p＜0.05. WT: wide type

Otherwise, we found that the *EGFR* mutation frequency in female patients was significantly higher than that in male (Figure 3c). While the frequency of mutations in *PTEN* was significantly lower in female than in male (Figure 3c). Next, we found that the frequency of mutations in *EGRR*, *MET*, *RET*, and *TP53* were significantly higher in nonsmoker patients than in smokers (Figure 3d). In addition, we correlated age with targetable genotype. We set age 55 as the cut‐off value for distinguishing young patients from old patients. A significant difference in the targeted genetic profile was found between the young and the older (Figure 4a, *p* = .0038). Notably, 91.9% (68/74) of young patients presented genetic mutations (*EGFR*, *ALK*, *ROS1*, *BRAF*, *KRAS*), compared with 82.6% (142/172) in the old patients. Among the gene mutations investigated, only *ALK* rearrangements (*p* = .00079) and *BRAF* (*p* = .0067) mutation genotypes were significantly associated with age at diagnosis, while mutations such as *EGFR*, *KRAS* and *MET* were no longer significantly associated with age (Figure 4b–f).

**Figure 5:**
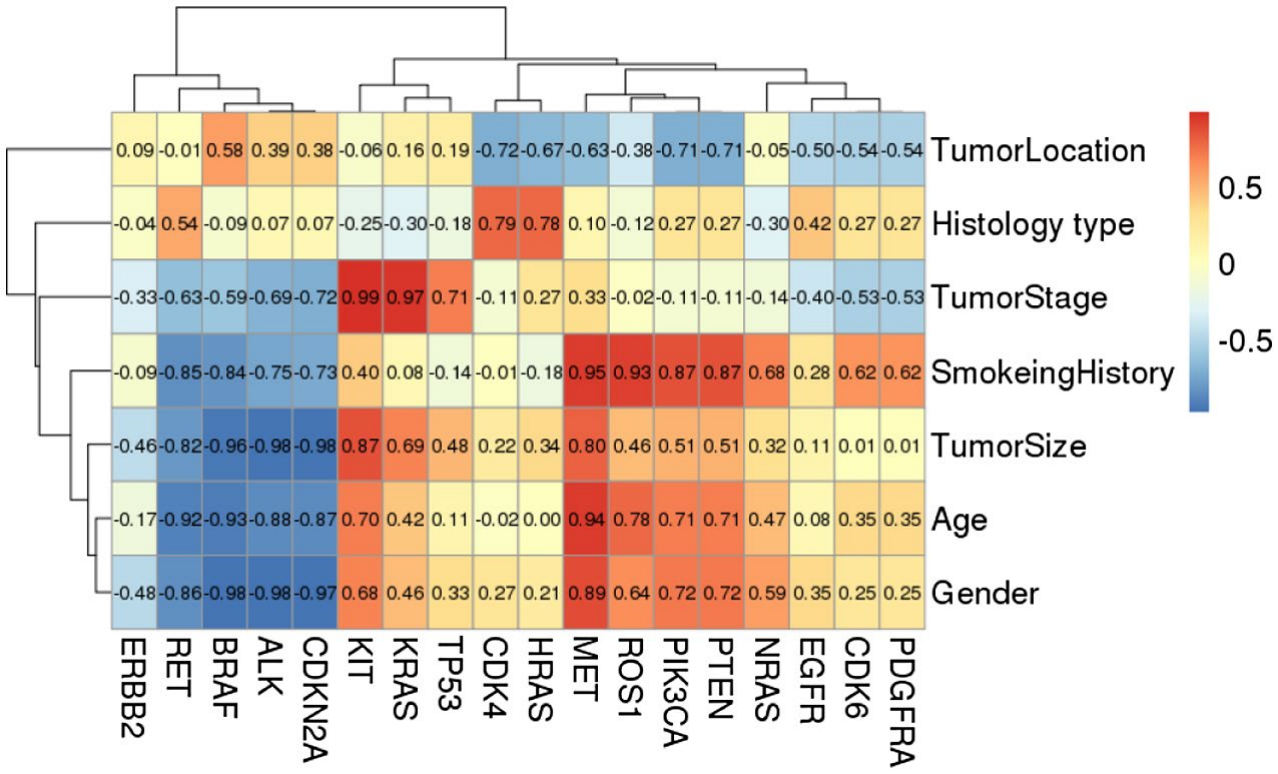
Pearson correlation analysis between gene mutations and clinical features. Positive number indicates positive correlation. Negative number indicates negative correlation. Correlation coefficients range from −1 to + 1

Figure 5 depicted a relationship between targeted genetic profile and clinical features. Among all tested genes, *KIT* (Pearson's *r* = .99) and *KRAS* (Pearson's r = 0.97) were positively correlated with patients’ age. In addition, smoking history was positively correlated with *MET*, *ROS1*, *PIK3CA*, and *PETN* gene mutations (Pearson's *r* = .87–.95) whereas *RET* (Pearson's *r* = −.85) and *BRAF* (Pearson's *r* = −.84) were negatively correlated. In addition, we found that several genes were highly correlated with age, such as *MET* (Pearson's *r* = .94), *ROS1* (Pearson's *r* = .78), *RET* (Pearson's *r* = −.92), *BRAF* (Pearson's *r* = −.93), *ALK* (Pearson's *r* = −.88), *CDNK2A* (Pearson's *r* = −.87). Notably, *BRAF* and *ALK* mutations in the younger and older groups were significantly different (Figure 4b,c). Moreover, we also found that *MET*, *RET*, *BRAF*, *AKL*, *CDKN2A* were highly correlated with gender, *MET* was positively correlated with female, and the remaining genes were positively correlated with male. The above results provided vital assistance in the diagnosis of NSCLC.

## 4 DISCUSSION

In the past decade, the treatment of cancer has gradually been evolving to targeted therapy and even immunotherapy. However, the effectiveness of targeted therapy depends on the gene mutation spectrum of tumor tissue (Hirsch, Suda, Wiens, & Bunn, 2016). As more and more studies have been conducted on targeted therapy, investigators have found that number of gene mutations associated with targeted therapy increase from one gene to several genes. Therefore, it is essential to conduct a comprehensive gene mutations profile analysis on tumor tissues (Kamps et al., 2017), rather than simply test a single gene. So far, the NCCN guidelines recommend a panel of genetic tests for NSCLC including *EGFR*, *ALK*, *ROS1*, *BRAF*, *MET*, *RET*, *ERBB2*, and *KRAS*. Multigene targeted sequencing is quick and economical lab test to provide a comprehensive spectrum of tumor tissue mutations. In the current study, we used targeted sequencing to comprehensively analyze the genetic profiles of 256 NSCLC patients. The top three driver gene mutations in NSCLC are *EGFR*, *ERBB2*, and *KRAS*. Of all patients, 63% (162/256) patients had multiple (two or more) mutations. Among the patients with two gene mutations, we found that one patient was co‐mutated with *EGFR* and *KRAS*. *KRAS* and *EGFR* mutations were usually mutually exclusive. Once they co‐exist, *KRAS* mutations might develop resistance to *EGFR* inhibitors (Pao et al., 2005). This finding suggested that this patient should be cautious when using EGFR‐TKI targeted therapy.

Wang et al analyzed the epidemic trend and pathological features of lung cancer in urban areas of Beijing from 1998 to 2007 (Wang et al., 2011). For tumor histological types, the proportion of squamous cell lung cancer is declining, while adenocarcinoma elevated especially in female patients. Alamoudi, (2010), Chang, Dai, Ren, Chen, and Guo (2012) and Cancer Genome Atlas Research Network (2012) also found the facts that the histological subtype of lung cancer changed from squamous carcinoma to adenocarcinoma. This explained why patients with adenocarcinoma (83.7%) were much more than squamous cell carcinoma (6.7%) in this study.

The average variant allele frequency (VAF) of adenocarcinoma was lower than that of squamous cell carcinoma, we speculated that the oncogenesis and development of squamous cell carcinoma were more closely related to genetic mutations than adenocarcinoma. In Asia, the most common gene mutation in adenocarcinoma patients is *EGFR*, and most of *EGFR* mutations occur in never smoker. In contrast, squamous cell lung cancer remains to be the most common subtype among severe smokers. To date, no targetable driver gene mutations have been found due to smoking, and the number of squamous cell histology mutations is higher than adenocarcinoma (2012; 2014). It was reported that the frequency of allele mutations in ctDNA was higher in metastatic or advanced stage cancer than in early stage cancer (Phallen & Sausen et al., 2017). Notably, our study focused on tumor tissues rather than ctDNA. Our results had first discovered that the average mutation frequency of stage I cancer was lower than that of advanced stage, we speculated that the accumulation of genetic mutations was associated with the evoluation of tumor clones. While the frequency of mutations in NSCLC‐associated driver genes is consistent with previous studies (Gou & Wu, 2014; Wang et al., 2011). Our results indicated that early NSCLC patients should also undergo genetic testing, which was beneficial to targeted treatment of patients.

Currently, nonsmoker lung adenocarcinoma is considered to be a unique disease due to its unique epidemiological, biological, and clinical characteristics. Many experts have named it as a unique entity (Sun et al., 2007; Yano et al., 2011). Many studies have shown that mutations in *EGFR* are associated with smoking status and specific histological types (Li et al., 2013; Ren et al., 2012; San Tam et al., 2006; Xu et al., 2012). A study of 506 cases of NSCLC showed that the mutation rate of *EGFR* was higher in nonsmoking patients than in smoking patients, and higher in female patients than in male patients (Wu et al., 2007). Another study of 524 patients with NSCLC also found that the rate of *EGFR* mutation vary with smoking status and histological subtypes, *EGFR* being the most frequently altered gene in nonsmoking adenocarcinoma patients (An et al., 2012). In this study, both smoking status and gender, the mutation rate of *EGFR* was consistent with previous studies.

Although young patients with nonsmall cell lung cancer account for about 1/20, more attention is being paid year by year (Arnold et al., 2016; Corrales‐Rodríguez et al., 2017; Thomas et al., 2015). Many studies have shown that young patients with NSCLC have unique characteristics: the incidence of NSCLC is higher in female, nonsmokers, and lung adenocarcinoma (Subramanian et al., 2010; Ye et al., 2014). A recent study of young patients with nonsmall cell lung cancer showed that higher frequency of *ALK* and *HER2* genetic alterations were associated with young age, while, mutations in *KRAS*, *STK11*, and *EGFR* exon 20 are more common in older patients (Hou et al., 2018). It is clear that we found a higher frequency of *ALK* mutations and a lower frequency of *KRAS* mutations in young patients (below 55) which is consistent with previous studies (Hou et al., 2018; Sacher et al., 2016; Tanaka et al., 2017). In addition, a higher frequency of *BRAF* mutations and a lower frequency of *MET* are also found in young patients. However, although *EGFR* is the most common mutation in nonsmall cell lung cancer and is more frequent in the elder patients, no significant differences are found between the young and old patients. Pearson correlation analysis determines the correlation between gene mutations and clinical features. Our results showed that *ALK*, *BRAF,* and *MET* mutations had a strong correlation with age. The above findings highlighted that the targeted alterations of *ALK* and *BRAF*, could be a valuable target in young patients with nonsmall cell lung cancer.

Our study has some limitations. First, this is a single center study with small sample size. Therefore, the results could be biased. Second, only a small panel of 18 genes was tested in this study and the findings were limited. In addition, although we described that there was a significantly higher EGFR mutation rate and other unique mutation features of cancer driver genes in the NSCLC patients, the majority of them were early stage (175 patients in stage I) which is still unnecessary to undergo targeted therapy. We expect a large panel of genetic tests for more discoveries in the future. And studies with a much larger sample size, for example multicenter trials, and longer duration of follow‐up are still necessary to confirm these results.

## CONFLICTS OF INTEREST

The authors have declared no conflicts of interest.

## AUTHOR CONTRIBUTION

Xianguo Chen: conceptualization, methodology, investigation; Bo Xu: collecting clinical data and samples; Qiang Li: data curation, formal analysis; Xiaoyi Xu: resources; Xianshuai Li: validation; Xia You: visualization, writing—original draft, writing—review & editing; Zhaonan Yu: validation, supervision, project administration, funding acquisition.
